# Exploring how entrepreneurship education and entrepreneurial mindset shape students’ entrepreneurial intention in an Iranian university context

**DOI:** 10.1371/journal.pone.0348903

**Published:** 2026-06-18

**Authors:** Saeid Karimi, Zahra Kosari Honarmand

**Affiliations:** Department of Agricultural Extension and Education, Bu-Ali Sina University, Hamedan, Iran; Shah Abdul Latif University, PAKISTAN

## Abstract

Despite the growing emphasis on entrepreneurship education, many university graduates continue to exhibit low entrepreneurial intention and engagement. Understanding how education and entrepreneurial mindset jointly influence this intention remains limited, particularly in non-Western contexts. Drawing on the Theory of Planned Behavior (TPB), this study investigates how entrepreneurship education and entrepreneurial mindset shape students’ entrepreneurial intention through the TPB constructs—attitude toward entrepreneurship, subjective norms, and perceived behavioral control (PBC). Using a self-administered survey, 190 valid responses from students at an Iranian public university were analyzed via structural equation modeling (SEM-PLS). The findings indicate that attitude and PBC significantly influence entrepreneurial intention, while subjective norms do not have a direct effect but exert an indirect influence through attitude and PBC. Entrepreneurship education positively affects PBC and indirectly influences entrepreneurial intention via PBC but does not directly predict intention, attitude, or subjective norms. Furthermore, entrepreneurial mindset significantly shapes attitude, subjective norms, and PBC, and indirectly influences entrepreneurial intention through these TPB components. Overall, the findings highlight the need for more experiential and practice-oriented educational approaches to ensure that improvements in entrepreneurial mindset and educational exposure translate into stronger entrepreneurial intention. Practical recommendations are offered to enhance the design and delivery of entrepreneurship education in higher education settings.

## Introduction

Entrepreneurship has emerged as a crucial driver of economic development, fostering innovation, generating employment, and enhancing societal well-being [1,2]. As economies transition toward knowledge-based and innovation-driven models, the role of entrepreneurial activity in addressing challenges such as unemployment and economic stagnation has gained significant scholarly and policy attention. Particularly in developing economies, where labor markets struggle to absorb the increasing number of graduates, entrepreneurship offers a viable alternative to traditional employment pathways [3]. In this context, universities are expected to nurture entrepreneurial competencies and cultivate graduates who are capable of creating new ventures rather than relying solely on traditional employment pathways [4].

At the heart of this agenda is entrepreneurial intentions, widely recognized as the strongest predictor of actual entrepreneurial behavior [5–7]. Entrepreneurial intention reflects an individual’s conscious willingness to initiate entrepreneurial action [8] and plays a decisive role in determining whether students consider entrepreneurship as a viable career path [9,10]. Understanding the factors that shape student entrepreneurial intention is therefore essential for strengthening entrepreneurial ecosystems within higher education and for designing more impactful educational interventions [2,10].

Ajzen’s Theory of Planned Behavior (TPB) [11] provides a robust and widely applied framework for explaining how entrepreneurial intention is formed. According to the TPB, entrepreneurial intention is shaped by three proximal antecedents: attitude toward entrepreneurship, subjective norms, and perceived behavioral control (PBC). These constructs have consistently emerged as strong predictors of entrepreneurial intention across diverse cultural and educational contexts [12,13]. Furthermore, the TPB allows for the inclusion of additional variables that can influence intention directly or indirectly through these core components, making it well-suited for integrating broader psychological or educational factors [11,14–16].

Two such predictors that have gained growing scholarly attention are entrepreneurship education and the entrepreneurial mindset. Entrepreneurship education seeks to develop students’ knowledge, skills, and attitudes related to entrepreneurial activity. Several studies suggest that exposure to entrepreneurship courses and training enhances students’ intention to start a business [17–21]. However, empirical findings on the effectiveness of entrepreneurship education in fostering entrepreneurial intention remain mixed, with some studies reporting insignificant or even negative effects [22–24]. These inconsistencies highlight the need for further research into the mechanisms through which education influences entrepreneurial intention [18,24].

The entrepreneurial mindset, characterized by creativity, resilience, risk tolerance, and opportunity recognition [25], has also been posited as a crucial psychological antecedent of entrepreneurial intention [26]. The entrepreneurial mindset is the individual’s determination to engage in entrepreneurial activities [27]. Individuals with a well-developed entrepreneurial mindset are more likely to perceive entrepreneurship as a viable career path, persist through challenges, and proactively seek out business opportunities [28, 29]. Despite its theoretical importance, limited research has explored how the entrepreneurial mindset interacts with the TPB framework or how it is shaped by entrepreneurship education [14, 19], particularly in non-Western contexts.

This study aims to bridge these gaps by examining how entrepreneurship education and entrepreneurial mindset shape students’ entrepreneurial intention through the components of the TPB. Grounded in the TPB, the proposed model positions entrepreneurship education as an external antecedent that influences students’ entrepreneurial mindset as well as their attitude, subjective norms, and perceived behavioral control—the proximal predictors of entrepreneurial intention. Specifically, it seeks to (1) the relationship between entrepreneurship education and the TPB components and, through them, entrepreneurial intention, (2) assess the effect of the entrepreneurial mindset on the TPB components and its indirect influence on entrepreneurial intention via these components, and (3) determine whether the entrepreneurial mindset mediates the relationship between entrepreneurship education and entrepreneurial intention.

By addressing these research questions, this study contributes to both theoretical and practical discussions on fostering entrepreneurship among students. Its findings will provide insights for educators, policymakers, and practitioners seeking to design more effective entrepreneurship education programs that cultivate not only business knowledge but also the mindset and cognitive structures necessary for entrepreneurial success.

## Theoretical framework and hypotheses

The TPB [11] provides a widely accepted framework for understanding entrepreneurial intention [13,30] with demonstrated validity [6]. It suggests that an individual’s decision to engage in entrepreneurship is driven by three key determinants: attitude toward entrepreneurship (ATE), subjective norms (SNs), and perceived behavioral control (PBC) [31]. These components shape entrepreneurial intention, which serves as a strong predictor of entrepreneurial behavior. Recent studies highlight the continued relevance of the TPB in predicting individuals’ intentions to start new business ventures [16,32].

Despite the TPB’s attempt to account for all factors influencing behavioral intentions, it has been criticized for overlooking important variables. Some scholars argue that the model lacks sufficient explanatory power to fully capture specific behaviors [2]. To address this limitation, researchers have suggested incorporating additional variables into the framework [16,33]. Ajzen [11] also highlights the TPB’s flexibility and its potential for extension, emphasizing that it is an open model in which external variables may influence intention indirectly through the three core antecedents.

**Fig 1:**
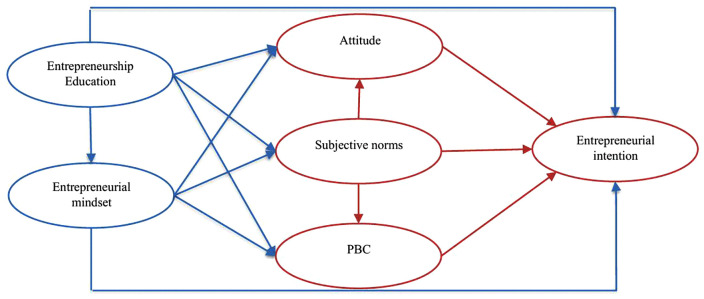
The Research conceptual model.

Drawing on this flexibility, the present study extends the TPB by integrating two key external factors: entrepreneurship education and the entrepreneurial mindset (Fig 1). Consistent with Social Cognitive Theory [34], entrepreneurship education is conceptualized as an environmental influence that shapes students’ cognitive processes. These cognitive changes foster the development of an entrepreneurial mindset, which subsequently affects students’ attitudes, subjective norms, and perceived behavioral control related to entrepreneurial behavior.

### Core TPB hypotheses

ATE reflects an individual’s positive or negative evaluation of becoming an entrepreneur. A favorable attitude increases the likelihood of forming strong entrepreneurial intention [13]. If individuals perceive entrepreneurship as a rewarding career path with beneficial outcomes, they are more inclined to pursue it [35]. Research has shown a strong association between ATE and entrepreneurial intention, indicating that students perceive entrepreneurship as an attractive and desirable career path. These findings suggest that, given the necessary opportunities and resources, students are likely to pursue entrepreneurial ventures [36–40].

SNs refer to the perceived social pressure to engage in entrepreneurial activities. These pressures come from influential figures such as family, friends, and mentors, shaping an individual’s entrepreneurial decision-making process [41]. If an individual perceives strong support from their social network, they are more likely to develop entrepreneurial intention [42]. While some studies have found a significant positive effect of SNs on entrepreneurial intention [40,43,44], others have found only a weak or insignificant relationship [2,45,46]. For this reason, Liñán and Santos [47] argue that SNs essentially represent a specific form of social capital and may, therefore, be related to the other two variables—ATE and PBC—transmitting their effect on entrepreneurial intention through these variables. When individuals perceive that their “reference people” approve of their decision to become entrepreneurs, they are more likely to be drawn to this option and feel more confident in their ability to pursue it successfully [47]. Therefore, SNs influence people’s evaluation about the effects and consequences of concerned behavior. Also, social pressure effectively affects people’s perception regarding external obstacles in terms of a specific behavior or its ease or difficulty. These relations have been also confirmed in different studies about entrepreneurial intention [48–50]. If individuals perceive positive social support for entrepreneurship, they are likely to develop a favorable attitude and believe in their ability to start a business, which in turn strengthen their entrepreneurial intention [39,51–53].

PBC represents an individual’s belief in their ability to successfully start and run a business. It encompasses confidence in entrepreneurial skills, resource availability, and the ability to overcome obstacles [54]. It is closely linked to self-efficacy, which represents confidence in one’s ability to overcome challenges in entrepreneurship [54,55]. Higher PBC leads to stronger entrepreneurial intentions, as individuals with greater self-efficacy are more likely to take action [56]. Lortie and Castogiovanni [57] verified that 90% of the articles analyzed in their review supported the positive relationship between perceived behavioral control and entrepreneurial intentions. Studies with university students also confirm a significant relationship between PBC and entrepreneurial intention [14,37,40].

Based on the above discussion, the following hypotheses are presented:

**H1a:** Attitude toward entrepreneurship is positively related to entrepreneurial intention.

**H1b:** Subjective norms are positively related to entrepreneurial intention.

**H1c**: Perceived behavioral control is positively related to entrepreneurial intention.

**H2:** Subjective norms are positively related to (a) attitude toward entrepreneurship and (b) perceived behavioral control.

**H3:** (a) Attitude toward entrepreneurship and (b) perceived behavioral control mediate the relationship between subjective norms and entrepreneurial intention.

### Extending the TPB: The role of entrepreneurship education

As summarized by Amofah and Saladrigues [58], entrepreneurship education is considered as any program, process or activities aimed at promoting entrepreneurial attitudes and skills. Entrepreneurship education is essential for shaping students’ entrepreneurial intention by providing them with the knowledge, skills, and motivation needed for self-employment [18,59,60]. Many graduates struggle to secure industry jobs despite possessing the necessary competencies [61], making entrepreneurship education a critical tool for fostering entrepreneurial careers.

#### Direct effect of entrepreneurship education on entrepreneurial intention.

According to the TPB, entrepreneurship education directly influences entrepreneurial intention and indirectly shapes it through key antecedents [62]. Empirical studies further support this view, demonstrating that entrepreneurship education significantly enhances students’ entrepreneurial intention [18,63,64]. Fishbein and Ajzen [65] argue that entrepreneurship education motivates learners to consider entrepreneurship as a viable career path by fostering self-determination, strengthening risk-management skills, and preparing them to recognize and pursue entrepreneurial opportunities. Similarly, Murad et al. [21] note that engagement in entrepreneurial learning activities increases students’ knowledge, capabilities, and likelihood of pursuing entrepreneurial careers, ultimately boosting their entrepreneurial intention. Consistent with this perspective, Martínez-Gregorio et al. [62] show that entrepreneurial intention can be cultivated when learners actively engage in business creation processes. Participation in real-world entrepreneurial activities—such as design thinking tasks and mastery experiences—enhances students’ confidence in their abilities and supports effective new venture creation. Likewise, Karimi et al. [17] found that students who take entrepreneurship courses or participate in related programs tend to exhibit higher levels of entrepreneurial intention. Based on this discussion, the following hypothesis is proposed:

**H4**: Entrepreneurship education is significantly and positively related to entrepreneurial intentions.

However, research on this relationship remains inconclusive. One explanation for these mixed findings is that entrepreneurship education is intertwined with other antecedents of entrepreneurial intention, such as ATE and PBC [24]. In addition, scholars argue that intervening factors influence this relationship, making it more complex than a straightforward cause-and-effect link [18]. These discrepancies highlight a critical gap in the literature, particularly within Asian contexts [66], and research conducted in Iran remains limited. To address this gap, the present study seeks to provide deeper insights into the role of entrepreneurship education in shaping entrepreneurial intentions.

#### Effects of entrepreneurship education on TPB components.

Entrepreneurship education not only expands students’ general knowledge of entrepreneurship but also shapes their perceptions of key components of the TPB—namely, ATE, SNs, and PBC [17,50]. According to Ajzen’s [11] theory, exogenous factors like entrepreneurship education influence entrepreneurial intention indirectly rather than directly. Scholars argue that entrepreneurship education enhances the TPB components by providing knowledge on resource accessibility, dispelling perceptions of infeasibility, and strengthening individuals’ belief in their ability to engage in entrepreneurship [39,45]. However, prior research presents mixed findings regarding its effects on the antecedents of entrepreneurial intention. Some studies report a positive influence on all three components [50,56,67–69], while others find a positive effect only on ATE and PBC [70–72]. Additionally, some research indicates a positive effect on ATE and/or SNs [45,73–75], while other studies highlight a positive impact on PBC and SNs [17,76].

Given these varying results, it is essential to examine these relationships among Iranian students to determine how entrepreneurship education influences their ATE, SNs, and PBC. Therefore, the following hypotheses are proposed:

**H5**: Entrepreneurship education is significantly and positively related to (a) attitude toward entrepreneurship, (b) subjective norms, and (c) perceived behavioral control.

#### Indirect effects of entrepreneurship education via TPB components.

The TPB components have been shown to mediate the relationships between various predictors—such as contextual factors [30] and cultural values [32]—and entrepreneurial intention. However, several recent studies argue that empirical evidence demonstrating the mediating role of TPB components in the relationship between entrepreneurship education and entrepreneurial intention remains limited [24]. Although some research suggests that the TPB components may mediate the effect of entrepreneurship education on entrepreneurial intention [15,70], findings remain inconclusive. Entrepreneurship education may positively shape students’ attitudes, strengthen perceived social support, and enhance their confidence in performing entrepreneurial tasks, which in turn could influence their intention to start a business. Thus, the following hypotheses are presented:

**H6a**: Attitude toward entrepreneurship mediates the relationship between entrepreneurship education and entrepreneurial intention.

**H6b**: Subjective norms mediate the relationship between entrepreneurship education and entrepreneurial intention.

**H6c**: Perceived behavioral control mediates the relationship between entrepreneurship education and entrepreneurial intention.

### Extending the TPB: The role of entrepreneurial mindset

The entrepreneurial mindset is a cognitive framework that enables individuals to recognize opportunities, take calculated risks, and persist in the face of challenges [25]. Research consistently supports its positive influence on entrepreneurial intention, as individuals with a strong entrepreneurial mindset are more likely to pursue entrepreneurial careers [14,28,77,78]. This mindset reflects an individual’s implicit intellectual abilities and motivation [79], influencing students’ inclination toward entrepreneurship. It also shapes entrepreneurial behavior by guiding individuals in creating value and taking action in real-world business scenarios [80]. Empirical studies highlight that key elements of an entrepreneurial mindset, such as creativity and innovation, positively impact entrepreneurial intention among university students [81,82]. Research conducted across different cultural contexts further reinforces this link. Studies on vocational students in Iran [83], university students in Taiwan [14], and college students in South Korea [84] consistently report a significant positive correlation between entrepreneurial mindset and entrepreneurial intention.

Originating from cognitive psychology [85], the concept of an entrepreneurial mindset has evolved to encompass critical and creative thinking [86]. Individuals with this mindset actively seek opportunities, develop innovative solutions, and navigate challenges with resilience [64,87,88]. Motivation plays a crucial role in this process, as people with an entrepreneurial mindset are more inclined to pursue entrepreneurial endeavors [78,89]. They perceive opportunities where others see risks, set ambitious goals, and persist in overcoming obstacles [90]. Additionally, this mindset fosters self-competence, problem-solving skills, and a proactive attitude toward entrepreneurship [91,92].

#### Direct effect of entrepreneurial mindset on entrepreneurial intention.

Empirical studies confirm the strong relationship between entrepreneurial mindset and entrepreneurial intention across diverse populations [26,64,93,94]. For instance, research in Saudi Arabia [26] and Indonesia [95] found that an entrepreneurial mindset significantly enhances students’ entrepreneurial intentions. Furthermore, Cui and Bell [78] demonstrated that entrepreneurship education strengthens this relationship by reinforcing cognitive and motivational factors based on Bandura’s Social Cognitive Theory [96]. These findings collectively suggest that fostering an entrepreneurial mindset increases the likelihood of individuals thinking and acting entrepreneurially, leading to the following hypothesis:

**H7:** The entrepreneurial mindset is significantly and positively related to entrepreneurial intention.

#### Effects of entrepreneurial mindset on TPB components.

The entrepreneurial mindset is expected to influence the key antecedents of entrepreneurial intention outlined in TPB. Having a good entrepreneurial mindset means equipping entrepreneurs with the necessary knowledge. This could be learned via their own company experience or gained through a shift in thinking in areas such as law, accounting, and management, among others. This knowledge will increase attitude toward entrepreneurship, which then leads them to choose appropriate business strategies [14]. Students with an entrepreneurial mindset are more likely to perceive entrepreneurship positively [39,78]. The entrepreneurial mindset is a way of thinking that guides behavior toward entrepreneurial activities and outcomes. It is a flexible and evolving state of mind influenced by an individual’s interactions with their environment [97]. Research highlights two key aspects of the entrepreneurial mindset: networking and resource leveraging [78]. Networking, viewed through a social capital lens, involves building and maintaining connections beyond one’s immediate circle, which is crucial for recognizing and utilizing entrepreneurial opportunities. Resource leveraging refers to accessing and utilizing resources that one does not own or control to achieve goals, playing a vital role in transforming ideas into action. Both networking and resource leveraging contribute to social support in entrepreneurship [14].

The entrepreneurial mindset fosters a sense of self-confidence and self-belief, which contributes to higher levels of entrepreneurial self-efficacy. When individuals develop an entrepreneurial mindset—characterized by adaptability, risk-taking, resilience, and opportunity recognition—they become more confident in their ability to start and manage ventures successfully [26,98]. Thus, on the basis of the given arguments, following hypotheses are postulated:

**H8**: The entrepreneurial mindset is significantly and positively related to (a) attitude toward entrepreneurship, (b) subjective norms, and (c) perceived behavioral control.

#### Indirect effects of entrepreneurial mindset via TPB components.

In addition to its direct effect on entrepreneurial intention, prior studies suggest that components of the Theory of Planned Behavior (TPB)—namely ATE, SN, and PBC—may also mediate the effect of entrepreneurial mindset on entrepreneurial intention. An entrepreneurial mindset can positively influence an individual’s beliefs, social perceptions, and perceived capability, thereby indirectly shaping their intention to engage in entrepreneurial activities. Thus, the following hypotheses are presented:

**H9a**: Attitude toward entrepreneurship mediates the relationship between entrepreneurial mindset and entrepreneurial intention.

**H9b**: Subjective norms mediate the relationship between entrepreneurial mindset and entrepreneurial intention.

**H9c**: Perceived behavioral control mediates the relationship between entrepreneurial mindset and entrepreneurial intention.

### Entrepreneurship education and entrepreneurial mindset

The entrepreneurial mindset is widely recognized as a key factor distinguishing entrepreneurs from non-entrepreneurs [99]. It is commonly defined as a way of thinking that emphasizes value creation despite resource constraints in uncertain and ambiguous environments [100]. This mindset enables individuals to recognize patterns among unrelated variables and transform them into innovative products or services, driving new venture creation or business growth [101]. Pidduck et al. [102] further refined this concept, defining the entrepreneurial mindset as a dispositional and opportunity-based schema that stimulates goal-oriented entrepreneurial behavior. Similarly, scholars have described it as the ability to act decisively and mobilize resources in uncertain conditions [103] or as a cognitive perspective that facilitates opportunity recognition, decision-making under limited information, and adaptability in complex situations [88]. Among these definitions, Daspit et al. [88] offer a comprehensive framework encompassing four key elements: risk propensity, dispositional optimism, ambiguity tolerance, and alertness to opportunity [101,104–106]. We adopt Daspit et al.‘s [88] definition as it fully integrates these essential components of an entrepreneurial mindset.

Entrepreneurship education plays a crucial role in shaping and strengthening the entrepreneurial mindset by fostering continuous learning, adaptability, and opportunity recognition [98]. Rooted in cognitive psychology, this perspective suggests that an entrepreneurial mindset is not innate but develops through prior knowledge and interactions with the environment [107]. At the metacognitive level, entrepreneurial thinking can be cultivated through education, reinforcing it as a learned habit [108]. It can be developed through targeted education and training, as mindset shifts occur through entrepreneurial learning [109]. Social Cognitive Theory provides a broad explanation for this relationship, emphasizing how environmental factors shape personal and behavioral variables [34]. In this framework, entrepreneurship education fosters an entrepreneurial mindset, which subsequently influences entrepreneurial intention. Empirical research highlights that entrepreneurship education enhances entrepreneurial abilities, attitudes, and motivation, equipping students with the skills necessary to identify and capitalize on opportunities [64,93,106,110]. Extracurricular programs further reinforce this mindset by encouraging experiential learning and practical engagement [104]. Despite extensive research on this topic, the relationship between entrepreneurship education and entrepreneurial mindset remains underexplored in developing countries, particularly in Iran. Based on this gap, the following hypothesis is proposed:

**H10:** Entrepreneurship education is significantly and positively related to the entrepreneurial mindset.

An entrepreneurial mindset enables individuals to internalize and apply the knowledge and skills gained through entrepreneurship education. It helps them grasp practical implications, recognize entrepreneurial opportunities, and act on them. This mindset also enhances self-efficacy, boosts confidence, and reduces fear of failure, thereby helping individuals navigate entrepreneurial challenges more effectively. Consequently, those with an entrepreneurial mindset are more likely to translate their entrepreneurial education into entrepreneurial intention [98]. Given evidence on the influence of entrepreneurship education on entrepreneurial mindset [104,110] and the impact of entrepreneurial mindset on intention [26,95], it is reasonable to expect that entrepreneurial mindset mediates the relationship between entrepreneurship education and entrepreneurial intention. This mediating role has been confirmed in studies among Chinese students [98,111]. Thus, the following hypothesis is presented:

**H11**: Entrepreneurial mindset mediates the relationship between entrepreneurship education and entrepreneurial intention.

## Research method

### Research design and sampling

This study employed a cross-sectional, quantitative research design using structural equation modeling with partial least squares (SEM-PLS) to test an extended TPB model. Data were collected via a self-administered survey in the Fall 2024. Prior research in entrepreneurship commonly uses undergraduate and graduate students as study participants, as they are typically highly motivated toward entrepreneurial activities [2,32,77]. In this study, the target population consisted of students from Bu-Ali Sina University, a major public university in western Iran. This university was purposively selected because it offers diverse academic programs (e.g., agriculture, humanities, engineering), ensuring heterogeneity in the sample; it has an established entrepreneurship curriculum, providing meaningful variation in exposure; and it provides logistical access that facilitated a high response rate.

A non-probability convenience sampling technique, stratified by faculty, was used due to accessibility and feasibility, while still ensuring adequate representation across disciplines. According to Hair et al. [112], the minimum sample size should be at least ten times the largest number of structural paths directed toward a single construct. In this model, entrepreneurial intention has seven incoming paths, requiring a minimum of 70 participants. To meet and exceed this requirement, 220 questionnaires were distributed, of which 200 were completed and returned (a 91% response rate). After removing incomplete responses, 190 questionnaires were retained for analysis.

### Measure

All scales used in this study were adapted from previous research. The survey tool was a closed-ended, researcher-developed questionnaire divided into two sections. The first section included demographic variables (e.g., age, gender), while the second section focused on the key variables in the model, such as entrepreneurial mindset, entrepreneurship education, and TPB constructs, which were measured using a series of items.

To measure the four variables of the TPB (i.e., entrepreneurial intention, ATE, SNs, and PBC), the questionnaire developed by Liñán and Chen [13] was used. The reliability of this questionnaire has been confirmed in various studies across different countries, including Iran [2,30,113]. The entrepreneurial intention scale included six items (e.g., “I am determined to create a business in the future.”). ATE was measured with five items (e.g., “A career as entrepreneur is attractive for me.”). SNs were assessed using three items (e.g., “If I decide to start a new business, my family members will support my decision.”). The PBC scale was measured with six items, which combined self-efficacy and controllability aspects (e.g., “To start a business and keep it working would be easy for me.”). Entrepreneurship education was measured using three items adopted from Wardana et al. [106], including the sample item: “Our university develops entrepreneurial skills.” The entrepreneurial mindset was measured using four items developed based on widely used scales in prior research [77,78], including the item: “I am constantly communicating with others to obtain new information.”

All items were rated on a 5-point Likert scale from 1 (“strongly disagree”) to 5 (“strongly agree”). The items were pre-tested with a small sample to ensure clarity and relevance. Before pre-testing, a panel of three academic experts in entrepreneurship reviewed the questionnaire to confirm content and face validity. Following their feedback, some items were revised. The survey was then pilot-tested with a convenience sample of 20 students. The results indicated satisfactory internal reliability, with all Cronbach’s alpha values exceeding the recommended threshold of 0.7 [112]. These pilot responses were excluded from the final dataset.

### Data analysis

SPSS version 27 was used for descriptive statistics and reliability analysis of the questionnaire, while SmartPLS version 4 was used for structural equation modeling (SEM-PLS) and hypothesis testing. This method was chosen because it is suitable for complex models, predictive analyses, and data that may deviate from normality or involve moderate sample sizes [112]. The analysis followed a two-stage approach: first, the measurement model was assessed for reliability, convergent validity, and discriminant validity. Second, the structural model was evaluated to test direct and indirect/mediating effects using bootstrapping with 5,000 subsamples. Path coefficients, significance levels, R², Q², and effect sizes (*f*²) were examined to assess relationships among entrepreneurship education, entrepreneurial mindset, TPB components, and entrepreneurial intention.

### Common method variance

Common method variance (CMV) was assessed because the data were collected through a self-report survey, which may introduce bias due to the use of a single measurement method. To mitigate this concern, both procedural and statistical remedies were applied [114]. Procedural remedies were incorporated into the ex-ante research design. Specifically, confidentiality and anonymity were assured to reduce social desirability bias, and survey items were carefully worded to be clear, specific, and free of leading or ambiguous language, thereby minimizing the likelihood of biased responses. For the ex-post assessment, Harman’s single-factor test was conducted [114]. The first unrotated factor accounted for 32.8% of the variance, which is well below the recommended 50% threshold. This indicates that common method variance was not a significant concern in this study.

### Ethics approval

This study was conducted in accordance with the ethical standards of Bu-Ali Sina University. The Research and Ethics Committee of the Department of Agricultural Extension and Education reviewed the study design and waived the requirement for formal ethics approval due to the anonymous and non-invasive nature of the survey. All participants provided informed consent prior to completing the questionnaire. Participation was voluntary, anonymity was ensured, and no identifying information was collected. All participants were college students over 18 years old and were informed about the study’s purpose and the intended use of the data. They had the right to participate voluntarily and to withdraw from the study at any time without providing a reason.

## Results

The results showed that 61.1% of the respondents were female, and 38.9% were male. The average age of participants was 21.18 years. In terms of academic discipline, 47.9% were enrolled in agricultural programs, 34.2% in humanities, and 17.9% in engineering. Furthermore, 88.9% were undergraduate students, 8.9% were pursuing a master’s degree, and 2.1% were doctoral students. About 23% of respondents had participated in entrepreneurship courses or programs, 56% personally knew one or more successful entrepreneurs, and 25% had work experience.

### Evaluation of the measurement model

To evaluate the measurement model, its reliability and validity were first examined. Reliability was assessed using Cronbach’s α and composite reliability (CR), while validity was evaluated through average variance extracted (AVE) for convergent validity and the Heterotrait-Monotrait (HTMT) ratio for discriminant validity. As shown in Table 1, the Cronbach’s alpha and CR values for all variables are above 0.7, indicating adequate reliability of the measurement model. Furthermore, Table 1 demonstrates that the AVE for all variables is greater than 0.5, confirming the model’s satisfactory convergent validity. According to Table 2, the HTMT values for all constructs are below the threshold of 0.85, indicating acceptable discriminant validity of the measurement model. Therefore, the evaluation of the measurement model confirms that all constructs exhibit appropriate reliability and validity.

**Table 1:** Analysis of measurement model results.

Constructs	Cronbach’s α	CR	AVE
**Entrepreneurial mindset**	0.73	0.83	0.55
**Entrepreneurship Education**	0.85	0.91	0.77
**Entrepreneurial intention**	0.91	0.93	0.69
**ATE**	0.91	0.93	0.69
**SNs**	0.82	0.89	0.74
**PBC**	0.89	0.92	0.65

**Table 2:** Assessment of discriminant validity using HTMT.

Constructs	1	2	3	4	5
**1-Entrepreneurial mindset**	–				
**2-Entrepreneurship Education**	0.40	–			
**3-Entrepreneurial intention**	0.47	0.26	–		
**4-ATE**	0.51	0.20	0.84	–	
**5-SNs**	0.43	0.22	0.52	0.58	–
**6-PBC**	0.58	0.45	0.68	0.57	0.53

### Evaluation of the structural model

The structural model was evaluated using key metrics, including R^2^ and Q^2^, and the significance of the hypothesized paths in the research framework. Model fit was assessed based on the strength of each structural path, as indicated by the R^2^ value for the dependent variable (exogenous construct), which should be at least 0.1. As shown in Table 3, the R^2^ values exceeded this threshold, confirming the model’s predictive capability. The predictive power of endogenous constructs was assessed using Q^2^, which should be greater than 0.000. The obtained Q^2^ values met the predefined criteria, ensuring strong predictive validity. Additionally, the supplementary model fit index, SRMR = 0.062, indicated a robust model fit for measuring each latent variable [112]. Next, hypothesis testing was conducted to assess the significance of the relationships.

**Table 3:** Analysis of predictive power and model fit.

Predictive power	Intention	ATE	SNs	PBC	Mindset
**R** ^**2**^	0.65	0.32	0.12	0.38	0.10
**Q** ^**2**^	0.43	0.23	0.08	0.24	0.06
**Model fit**					
**SRMR**	0.062				

The structural model was validated by reporting path coefficient (*β*), *p*-values, effect size (*f*^*2*^), and *t*-values, using a bootstrapping approach with 5,000 sub-samples, as recommended by Hair et al. (2019). Table 4 presents the analysis results, indicating that all hypotheses were supported except for H1b, H4, H5a, H5b, H7, H6a, H6b, H9b, and H11.

**Table 4:** Analysis of structural model results.

Hypotheses	Relationship	*β*	SD	*t-*value	*p-*value	Decision
Direct effect						
H1a	ATEàIntention	0.60	0.06	9.25	0.000	Supported
H1b	SNsàIntention	0.01	0.06	0.23	0.821	Unsupported
H1c	PBCàIntention	0.31	0.06	4.82	0.000	Supported
H2a	SNsàAttitude	0.40	0.09	4.32	0.000	Supported
H2b	SNsàPBC	0.32	0.08	4.05	0.000	Supported
H4	Entrepreneurship EducationàIntention	0.01	0.05	0.10	0.901	Unsupported
H5a	Entrepreneurship EducationàATE	0.01	0.07	0.12	0.921	Unsupported
H5b	Entrepreneurship EducationàSNs	0.09	0.08	1.20	0.199	Unsupported
H5c	Entrepreneurship EducationàPBC	0.24	0.06	3.82	0.000	Supported
H7	MindsetàIntention	−0.02	0.06	0.32	0.322	Unsupported
H8a	MindsetàATE	0.29	0.08	3.43	0.001	Supported
H8b	MindsetàSNs	0.30	0.06	4.15	0.000	Supported
H8c	MindsetàPBC	0.30	0.07	4.45	0.000	Supported
H10	Entrepreneurship EducationàMindset	0.32	0.07	4.35	0.000	Supported
Indirect Effects						
H3a	SNsàATEàIntention	0.24	0.06	4.10	0.000	Supported
H3b	SNsàPBCàIntention	0.10	0.03	3.30	0.001	Supported
H6a	Entrepreneurship EducationàATEàIntention	0.001	0.03	0.12	0.979	Unsupported
H6b	Entrepreneurship EducationàSNsàIntention	0.001	0.01	0.17	0.863	Unsupported
H6c	Entrepreneurship EducationàPBCàIntention	0.07	0.03	2.87	0.004	Supported
H9a	MindsetàATEàIntention	0.17	0.05	3.29	0.001	Supported
H9b	MindsetàSNsàIntention	0.01	0.02	0.21	0.831	Unsupported
H9c	MindsetàPBCàIntention	0.09	0.03	3.14	0.002	Supported
H11	Entrepreneurship EducationàMindset àIntention	−0.01	0.02	0.31	0.755	Unsupported
Total effect						
	SNsàIntention	0.35	0.09	4.05	0.000	
	Entrepreneurship EducationàIntention	0.23	0.07	3.26	0.001	
	MindsetàIntention	0.35	0.07	4.87	0.000	

Regarding the cognitive factors influencing entrepreneurial intention, attitude toward entrepreneurship had a significant impact (β = 0.60, *f*^*2*^ = 0.66, *t* = 9.25, *p* < 0.001)., confirming H1a. However, SNs did not have a significant effect on entrepreneurial intention (*β* = −0.01, *f*^*2*^ = 0.001, *t* = 0.23, *p* > 0.05)., leading *t*o the rejection of H1b. Additionally, PBC was found to significantly influence entrepreneurial intention (*β* = 0.31, *f*^*2*^ = 0.14, *t* = 4.82, *p* < 0.001), confirming H1c. Additionally, SNs posi*t*ively influenced attitude toward entrepreneurship (*β* = 0.40, *f*^*2*^ = 0.25, *t* = 4.32, *p* < 0.001), supporting H2a. SNs also had a positive effec*t* on PBC (*β* = 0.32, *f*^*2*^ = 0.14, *t* = 4.05, *p* < 0.001), confirming H2b.

Regarding the relationship between entrepreneurship education and the TPB constructs, entrepreneurship education positively influenced PBC (*β* = 0.24, *f*^*2*^ = 0.09, *t* = 3.82, *p* < 0.001), confirming H5c. However, its effects on entrepreneurial intention (*β* = 0.01, *f*^*2*^ = 0.000, *t* = 0.90, *p* > 0.05), ATE (*β* = −0.01, *f*^*2*^ = 0.000, *t* = 0.92, *p* > 0.05), and SNs (*β* = −0.09, *f*^*2*^ = 0.01, *t* = 01.20, *p* > 0.05) were no*t* significant, leading *t*o the rejection of H4, H5a, and H5b. These findings indicate that entrepreneurship education indirectly influences entrepreneurial intention via PBC. Meanwhile, the relationship between entrepreneurship education and entrepreneurial mindset was significant (*β* = 0.32, *f*^*2*^ = 0.10, *t* = 4.35, *p* < 0.001), confirming H10.

Regarding the effect of entrepreneurial mindset on the TPB components, its association with entrepreneurial intention was not significant (*β* = −0.02, *f*^*2*^ = 0.000, *t* = 0.32), leading to the rejection of H7. However, entrepreneurial mindset significantly influenced ATE (*β* = 0.29, *f*^*2*^ = 0.07, *t* = 3.43, *p* < 0.01), confirming H8a. Additionally, entrepreneurial mindset had a significant positive effect on SNs (*β* = 0.30, *f*^*2*^ = 0.10, *t* = 4.15, p < 0.001), confirming H8b. Similarly, en*t*repreneurial mindset positively influenced PBC (*β* = 0.30, *f*^*2*^ = 0.12, *t* = 4.45, *p* < 0.001), supporting H8c.

To examine the indirect effects among the research variables, a bootstrap test with 5,000 samples at 95% confidence intervals (CI) was conducted, following the guidelines of Preacher and Hayes [115]. As shown in Table 4, SNs had a significant indirect effect on entrepreneurial intention through both ATE (*β* = 0.24, *t* = 4.10, *p* < 0.001, [0.145, 0.382]) and PBC (*β* = 0.10, *t* = 3.30, *p* < 0.01, [0.039, 0.148]), suppor*t*ing H3a and H3b. Entrepreneurship education showed a significant indirect effect on entrepreneurial intention only through PBC (*β* = 0.07, *t* = 2.87, *p* < 0.01, [0.030, 0.127]), confirming H6c. However, its indirec*t* effects via attitude (*β* = 0.001, *t* = 0.979, *p* > 0.05, [−0.078, 0.078]), SNs (β = 0.001, *t* = 0.863, *p* > 0.05, [−0.022, 0.013]), and entrepreneurial mindse*t* (β = −0.01, *t* = 0.755, *p* > 0.05, [−0.012, 0.059]) were no*t* significant, thus H6a, H6b, and H11 were not suppor*t*ed. Regarding entrepreneurial mindset, significant indirect effects on entrepreneurial intention were observed via ATE (*β* = 0.17, *t* = 3.29, *p* < 0.01, [0.054, 0.243]) and PBC (*β* = 0.09, *t* = 3.14, *p* < 0.01, [0.038, 0.147]), confirming H9a and H9c. However, the mediation via SNs was not significant (*β* = 0.01, *t* = 0.831, *p* > 0.05, [−0.049, 0.035]), thus H9b was no*t* supported.

Overall, twenty-three hypotheses—including direct and indirect effects—were tested in the structural model. Of these, 14 were supported and 9 were not supported, indicating strong but selective relationships among the variables. Consistent with the TPB, ATE and PBC emerged as the strongest predictors of entrepreneurial intention, with large and medium effect sizes respectively, while SNs showed no direct effect. Entrepreneurship education did not directly predict intention or ATE/SNs and showed only a small effect on PBC, indicating that its influence operates mainly indirectly. The entrepreneurial mindset also showed meaningful effects on ATE, SNs, and PBC, although its direct impact on intention was nonsignificant. Overall, the findings highlight that TPB components—particularly ATE and PBC—remain the primary drivers of entrepreneurial intention, while education and mindset shape intention primarily through their indirect effects on the TPB antecedents.

## Discussion

This study explores the relationships between entrepreneurship education, the entrepreneurial mindset, and the components of the Theory of Planned Behavior (TPB) in shaping Iranian students’ entrepreneurial intentions. The findings highlight the crucial role of cognitive and educational factors in fostering entrepreneurship among university students. Notably, attitude toward entrepreneurship (ATE) and perceived behavioral control (PBC) emerged as significant predictors of entrepreneurial intention, confirming H1a and H1c, while subjective norms (SNs), contrary to H1b, had no direct effect. These results suggest that students’ confidence in their entrepreneurial abilities and their perception of entrepreneurship as a desirable career path play a more decisive role than external social pressures.

These findings align with prior research highlighting the importance of ATE and PBC in shaping entrepreneurial intention [14,24,50]. The strong effect of ATE supports studies suggesting that individuals with a favorable perception of entrepreneurship are more likely to pursue entrepreneurial careers [36]. Similarly, the significant role of PBC aligns with Bandura’s [55] self-efficacy theory, which posits that confidence in one’s ability to overcome entrepreneurial challenges fosters entrepreneurial intention [54]. Iranian students’ entrepreneurial intention appears to be driven more by individual considerations rather than normative or social influences [2]. Therefore, the current findings support prior studies suggesting that SN are the least influential determinant of entrepreneurial intention within the TPB framework [2,24]. However, consistent with H2a, H2b, H3a, and H3b, SNs were found to exert significant indirect effects on entrepreneurial intention through attitude and PBC. This interpretation aligns with previous research indicating that SNs primarily influence intention through shaping individuals’ beliefs and perceptions [24,39,53]. Thus, although SNs do not directly motivate Iranian students to start a business, they do influence the cognitive mechanisms that ultimately explain entrepreneurial intention.

The relationship between entrepreneurship education and the constructs of the TPB produced mixed insights. While entrepreneurship education significantly influenced PBC (supporting H5c), its effects on entrepreneurial intention (rejecting H4), attitude toward entrepreneurship (rejecting H5a), and SNs (rejecting H5b) were not significant. This contrasts with some previous studies reporting positive effects on attitude and intention [17–19]. Duong [24] also reported that entrepreneurship education can enhance students’ business-related knowledge and experience, foster entrepreneurial motivation and self-efficacy, and ultimately increase the likelihood of engaging in entrepreneurial activities. A possible explanation relates to the nature and structure of entrepreneurship education in public universities in Iran. Programs tend to emphasize theoretical knowledge rather than hands-on entrepreneurial experiences, creativity training, or real-world business exposure. As a result, while students gain knowledge and perceive themselves as more capable (hence the effect on PBC), their attitudes and motivations may not shift meaningfully because they lack practical engagement, mentorship, or experiential learning activities. Without these elements, education may not be powerful enough to reshape deeply held beliefs or generate genuine entrepreneurial enthusiasm.

Another explanation is that entrepreneurship is generally perceived as risky and uncertain in Iran’s economic context. Even if education increases skills, students may remain reluctant to express entrepreneurial intention due to financial insecurity, limited funding opportunities, and uncertain market conditions. Moreover, cultural preferences for secure and stable employment may override the influence of educational inputs, thereby preventing meaningful attitudinal change despite improved competencies. Thus, structural and contextual barriers may prevent education from influencing attitudes or intention directly. Nevertheless, education influenced intention indirectly through PBC (supporting H6c), consistent with theoretical expectations that the TPB mediates exogenous influences [50,62]. This reinforces the idea that educational interventions must strengthen both competency and motivation to impact intention effectively.

The results indicated that entrepreneurship education significantly enhances students’ entrepreneurial mindset (supporting H10), aligning with prior research highlighting its role in fostering entrepreneurial thinking and behavior [64,93,106,110]. However, despite this increase in mindset, entrepreneurial intention was not directly influenced by the entrepreneurial mindset (rejecting H7). While a strong entrepreneurial mindset enhances students’ attitudes, perceptions of social support, and self-efficacy (supporting H8a–H8c) [39], it may not be sufficient on its own to trigger entrepreneurial intention. A plausible explanation is that while an entrepreneurial mindset equips students with opportunity-recognition skills, creativity, and proactive thinking, these cognitive dispositions alone may be insufficient to form concrete entrepreneurial intention without the presence of enabling conditions. In the Iranian context—where economic uncertainty, limited access to financial resources, and strong preferences for stable employment persist—students may develop entrepreneurial thinking yet remain reluctant to commit to entrepreneurial careers. Thus, the mindset appears to function primarily by shaping beliefs and self-efficacy, which then influence intention indirectly rather than producing immediate motivational outcomes. This pattern aligns with prior research suggesting that the entrepreneurial mindset enhances cognitive readiness but requires additional motivational, contextual, or experiential triggers to convert into explicit entrepreneurial intention [14,78].

### Theoretical implications

This study provides several contributions to entrepreneurship theory. First, it provides empirical support for expanding the TPB model by incorporating cognitive constructs such as the entrepreneurial mindset. This expansion enriches Ajzen’s [11] framework by highlighting how metacognitive factors shape entrepreneurial intention through indirect pathways. The results suggest that entrepreneurial mindset plays a crucial role in influencing the key TPB components—ATE, SNs, and PBC—rather than directly affecting entrepreneurial intentions. This underscores the importance of recognizing the indirect cognitive pathways through which mindset shapes entrepreneurial intentions. The results therefore highlight the need for theoretical models that capture the nuanced role of metacognition and opportunity-oriented thinking in entrepreneurial decision-making. Furthermore, the study reinforces the central mediating role of PBC in translating educational experiences into entrepreneurial intention. While entrepreneurship education did not directly affect intention or attitude, its strong effect on PBC indicates that students’ confidence and perceived capabilities serve as key mechanisms linking education to intention. Theoretically, this emphasizes the importance of integrating self-efficacy [55] and resilience perspectives into the TPB to better understand how educational and cognitive factors jointly drive entrepreneurial motivation.

### Practical implications

From a practical perspective, the findings suggest that entrepreneurship education programs should focus on enhancing students’ PBC and entrepreneurial mindset rather than merely promoting entrepreneurship as a career choice. Given that entrepreneurship education did not directly influence entrepreneurial intention or attitude in this study, one important implication is the need to redesign educational approaches to strengthen these non-significant pathways. Universities and policymakers should co-design hybrid experiential learning models that combine theoretical instruction with hands-on entrepreneurial activities. Such models may include business simulations, venture creation projects, case-based learning, entrepreneurial internships, and applied community-based assignments. These experiential components help students translate learned knowledge into confidence and motivation—key mechanisms that can strengthen the direct impact of education on entrepreneurial intention.

Integrating structured mentorship programs is equally crucial. Mentorship connects students with experienced entrepreneurs who can provide practical guidance, social support, and real-world insights. This personal engagement can inspire students, reduce fear of failure, and help them internalize entrepreneurial attitudes, thereby reinforcing the pathways that were non-significant in the present study. Embedding mentorship into coursework or linking students with incubator-based mentors can accelerate mindset development and intention formation. Curricula should also go beyond theoretical instruction to incorporate practical activities that cultivate real-world entrepreneurial skills, equipping students with the resources, confidence, and capabilities needed to start and manage a business. The integration of life-based learning models, such as those proposed by Yoto et al. [116], is particularly valuable. These models—featuring entrepreneur success stories, company visits, and firsthand exposure to business development—can deepen students’ entrepreneurial mindset and stimulate intention formation. Schools should actively engage successful entrepreneurs to share their experiences, offering students inspiration, social validation, and realistic expectations about entrepreneurial careers.

Additionally, targeted interventions that nurture an entrepreneurial mindset—such as creativity workshops, resilience training, and opportunity recognition exercises—may indirectly contribute to entrepreneurial intention by strengthening attitudinal and self-efficacy beliefs. Given the significant role of entrepreneurial mindset in shaping cognitive antecedents, universities should prioritize courses and learning environments that encourage critical thinking, opportunity exploration, creativity, and risk-taking. Furthermore, collaboration between policymakers and the private sector can expand students’ exposure to entrepreneurial ecosystems. Access to incubators, accelerators, startup events, and networking activities can bridge the gap between education and practice, providing students with meaningful opportunities to apply their knowledge. Finally, entrepreneurship education should be tailored to diverse student backgrounds and academic disciplines, recognizing that different fields may require unique pedagogical approaches to fostering entrepreneurial intentions.

### Limitations and future research directions

While this study offers valuable insights, it has certain limitations. First, the cross-sectional design limits causal inferences. Most research in this field relies on cross-sectional data, which restricts the ability to establish causal relationships. Future studies should employ longitudinal designs to examine the dynamic relationships between the study variables over time, providing stronger evidence of causality. Second, data were collected through a self-reported questionnaire, which may introduce biases such as social desirability bias, where participants provide responses they believe are expected rather than reflecting their true intentions. Although steps were taken to ensure anonymity and confidentiality, future studies could use experimental designs or include objective measures of entrepreneurial behavior to reduce this bias. Third, the study sample was drawn from a single university in Iran, limiting the generalizability of the findings. Future research should include samples from multiple universities and countries to enhance the external validity of the results.

In addition to these methodological limitations, future research should explore how gender-based differences influence entrepreneurial intention formation. Given cultural and economic dynamics in Iran and other developing contexts, gender norms may affect how students internalize entrepreneurship education, develop an entrepreneurial mindset, and translate cognitive perceptions into intentions. Examining whether gender moderates the relationships among the TPB components, education, mindset, and entrepreneurial intention could provide a more nuanced understanding of entrepreneurial motivation. Finally, future research should investigate the role of contextual factors—such as economic conditions, cultural values, and institutional support—in shaping the relationships between entrepreneurship education, entrepreneurial mindset, and entrepreneurial intention. Cross-country comparisons and mixed-method approaches could offer deeper insights into how these factors contribute to differences in entrepreneurial intention across diverse settings.

## Conclusion

This study advances the understanding of how entrepreneurship education and cognitive factors interact to shape students’ entrepreneurial intentions. The findings indicate that attitude toward entrepreneurship and perceived behavioral control are the strongest direct predictors of entrepreneurial intention, whereas entrepreneurship education and entrepreneurial mindset influence intention primarily through these cognitive antecedents rather than through direct effects. This highlights the importance of fostering an entrepreneurial mindset and enhancing students’ confidence in their capabilities as mechanisms for indirectly shaping entrepreneurial intention. The results underscore the need for a holistic approach to entrepreneurship education that goes beyond theoretical instruction to include experiential learning, mentorship, and practical skill development. By strengthening students’ cognitive foundations and self-efficacy, universities can more effectively promote entrepreneurial behavior, ultimately contributing to the cultivation of a new generation of capable entrepreneurs and supporting broader economic development.

## Supporting information

S1 File Data. (XLS)
